# d-Allulose Ameliorates Skeletal Muscle Insulin Resistance in High-Fat Diet-Fed Rats

**DOI:** 10.3390/molecules26206310

**Published:** 2021-10-19

**Authors:** Yang Gou, Bingyang Liu, Mengyao Cheng, Takako Yamada, Tetsuo Iida, Sixian Wang, Ryoichi Banno, Teruhiko Koike

**Affiliations:** 1 Department of Sports Medicine, Graduate School of Medicine, Nagoya University, Nagoya 464-8601, Aichi, Japan; gou.yang@i.mbox.nagoya-u.ac.jp (Y.G.); byliu92@163.com (B.L.); wangsixiansichuan@163.com (S.W.); ryouichi@med.nagoya-u.ac.jp (R.B.); 2 Undergraduate School of Medicine, Nagoya University, Nagoya 464-8601, Aichi, Japan; pen365m@gmail.com; 3 Research and Development, Matisutani Chemical Industry Co., Ltd., Itami 664-8508, Hyogo, Japan; takako-yamada@matsutani.co.jp (T.Y.); tetsuo-iida@matsutani.co.jp (T.I.); 4 Research Center of Health, Physcal Fitness and Sports, Nagoya University, Nagoya 464-8601, Aichi, Japan

**Keywords:** d-allulose, insulin resistance, hyperinsulinemic–euglycemic clamp, white adipose tissue, inflammation, skeletal muscle, glucose uptake

## Abstract

Background: d-Allulose is a rare sugar with antiobesity and antidiabetic activities. However, its direct effect on insulin sensitivity and the underlying mechanism involved are unknown. Objective: This study aimed to investigate the effect of d-allulose on high-fat diet (HFD)-induced insulin resistance using the hyperinsulinemic–euglycemic (HE)-clamp method and intramuscular signaling analysis. Methods: Wistar rats were randomly divided into three dietary groups: chow diet, HFD with 5% cellulose (HFC), and HFD with 5% d-allulose (HFA). After four weeks of feeding, the insulin tolerance test (ITT), intraperitoneal glucose tolerance test (IPGTT), and HE-clamp study were performed. The levels of plasma leptin, adiponectin, and tumor necrosis factor (TNF)-α were measured using the enzyme-linked immunosorbent assay. We analyzed the levels of cell signaling pathway components in the skeletal muscle using Western blotting. Results: d-allulose alleviated the increase in HFD-induced body weight and visceral fat and reduced the area under the curve as per ITT and IPGTT. d-Allulose increased the glucose infusion rate in the two-step HE-clamp test. Consistently, the insulin-induced phosphorylation of serine 307 in the insulin receptor substrate-1 and Akt and expression of glucose transporter 4 (Glut-4) in the muscle were higher in the HFA group than HFC group. Furthermore, d-allulose decreased plasma TNF-α concentration and insulin-induced phosphorylation of stress-activated protein kinase/Jun N-terminal kinase in the muscle and inhibited adiponectin secretion in HFD-fed rats. Conclusions: d-allulose improved HFD-induced insulin resistance in Wistar rats. The reduction of the proinflammatory cytokine production, amelioration of adiponectin secretion, and increase in insulin signaling and Glut-4 expression in the muscle contributed to this effect.

## 1. Introduction

Insulin resistance occurs when tissues, including the skeletal muscle, adipose tissue, and liver, do not respond well to insulin [1] and is responsible for obesity, hyperglycemia, hyperlipidemia, and hypertension. Insulin resistance is a global health problem [2]. Insulin resistance can develop because of one or more of the following factors: overweight or obesity [3], Western-style diet [4], and sedentary lifestyle [5].

d-Allulose, C-3 epimer of fructose, has a molecular formula of C_6_H_12_O_6_. It is odorless and highly soluble in water and appears as a white crystalline substance. d-Allulose has approximately 70% relative sweetness and 90% calorie reduction compared with sucrose. The United States Food and Drug Administration has confirmed d-allulose as a generally recognized as safe substance, which can be used for food and dietary supplement [6]. Studies on the effect of d-allulose on insulin resistance have focused on the following areas: (1) increasing β cell function in the pancreas [7]; (2) promoting hepatic glucokinase translocation to increase hepatic glycogenesis [8]; and (3) suppression of lipid accumulation and inflammatory response in adipose tissue [9]. However, studies on the skeletal muscle, a critical tissue responsive to insulin, are lacking.

To our knowledge, this is the first report on the effect of d-allulose on insulin resistance in a high-fat diet (HFD)-induced obese model using the hyperinsulinemic–euglycemic (HE)-clamp method, which is the gold standard for assessing insulin resistance. The two-step HE-clamp analysis can reveal the effect of d-allulose on systemic and skeletal muscle insulin resistance. Through the HE-clamp method, our team confirmed that d-allulose could improve high sucrose-induced insulin resistance [10]. About the high-fat diet model, we investigated the role of d-allulose on insulin resistance from the perspective of insulin signaling in skeletal muscle and levels of inflammatory cytokines and adipokines derived from adipose tissue. Our research may provide the necessary evidence for developing d-allulose to prevent obesity-related insulin resistance.

## 2. Results

### 2.1. d-Allulose Supplementation Suppresses Increase in Body Weight and White Adipose Tissue Weights

**Figure 1:**
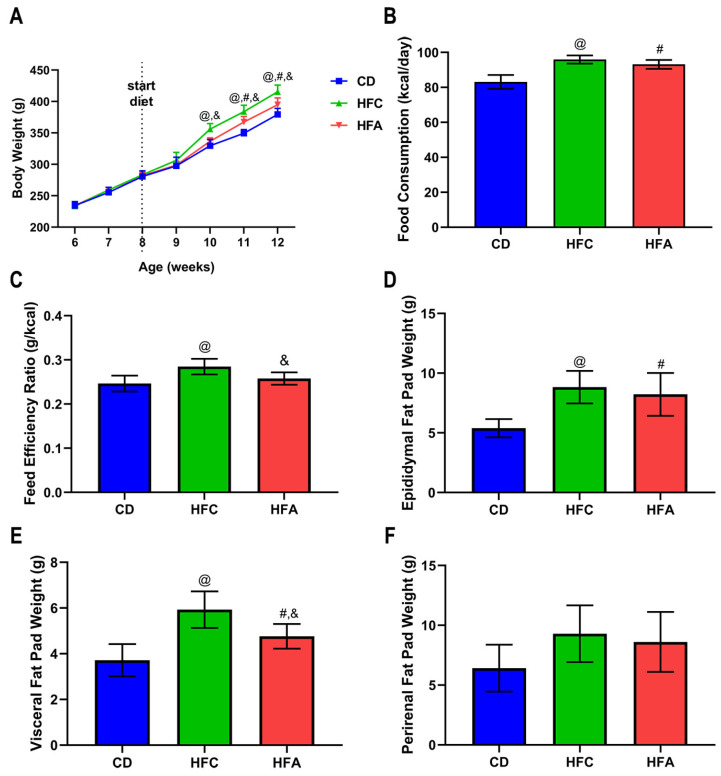
Effects of d-allulose supplementation for 4 weeks on body composition and calorie intake. (**A**) Bodyweight, (**B**) food consumption, and (**C**) feed efficiency ratio. (**D**) Epididymal, (**E**) perirenal, and (**F**) visceral fat pad weights. Results are expressed as mean ± SD. @: CD vs. HFC, #: CD vs. HFA, &: HFC vs. HFA. *p* < 0.05, n = 6 per group; ns, no significant difference. The differences were determined using one-way ANOVA (**A**–**F**). Skeletal muscle masses were presented in Supplementary Table S1. CD: chow diet, HFA: HFD + d-allulose, HFC: HFD + cellulose, and HFD: high-fat diet.

The body weights of the HFC group were higher than those of the chow diet (CD) group and HFA groups after 10 weeks old of the rats; between the HFC group and the HFA group, d-allulose supplementation produced around 5% weight loss (Figure 1A). The mean calorie intake of the CD group (83.2 ± 3.9 kcal/day) was significantly lower than the other two groups, but there was no significant difference between the HFC group (95.9 ± 2.4 kcal/day) and the HFA group (93.2 ± 2.5 kcal/day) (Figure 1B). Compared with the CD group, the HFC group had a higher feed efficiency ratio (FER). However, the HFA group had a lower FER than the HFC group (Figure 1C).

The epididymal and visceral fat in the HFC group were higher than those in the CD group (Figure 1D,E), whereas the HFA group had lower visceral adipose tissue weight compared to the HFC group (Figure 1E). There were no differences among the groups of perirenal fat weight (Figure 1F).

### 2.2. d-Allulose Improves Glucose Metabolism and Insulin Sensitivity

**Figure 2:**
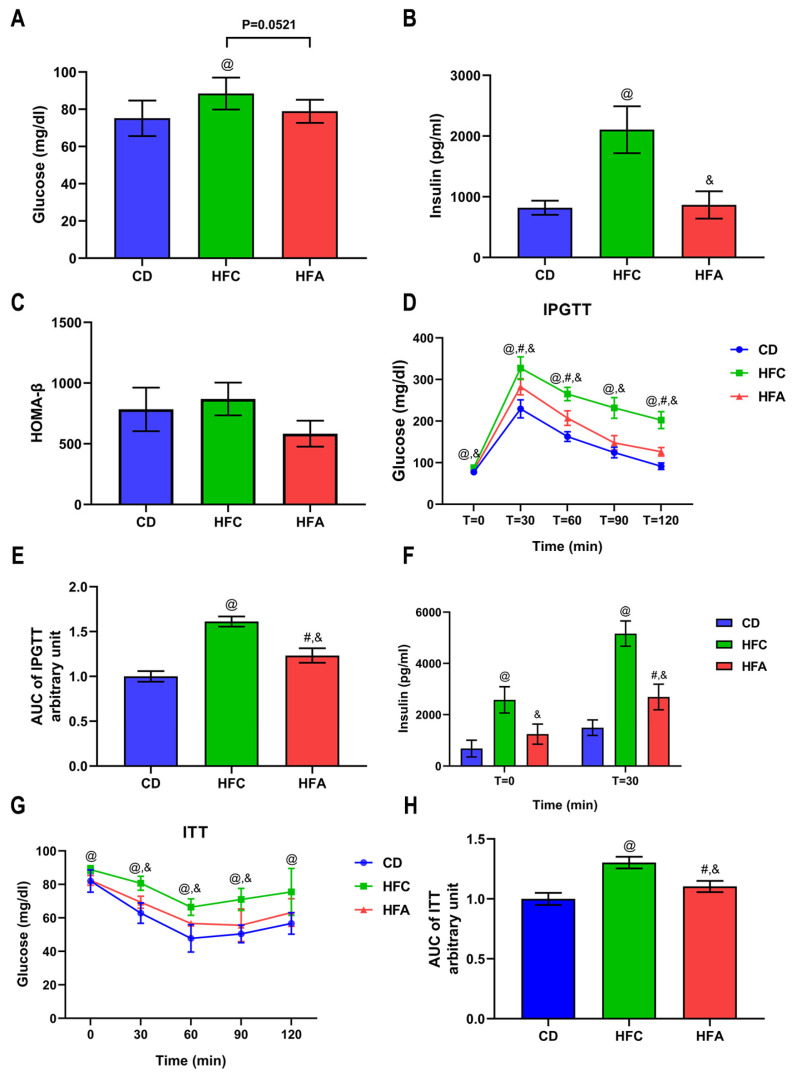
Effects of d-allulose supplementation for 4 weeks on glucose metabolism. (**A**) Fasting blood glucose level, (**B**) fasting plasma insulin level, (**C**) the value of HOMA-β, (**D**) blood glucose levels during IPGTT, (**E**) area under the curve during IPGTT, (**F**) insulin level at T = 0 and T = 30 min during IPGTT, (**G**) blood glucose levels during ITT, and (**H**) area under the curve during ITT. Results are expressed as mean ± SD. @: CD vs. HFC, #: CD vs. HFA, &: HFC vs. HFA, *p* < 0.05, n = 6 per group; and ns, no significant difference. Differences were determined using two-way ANOVA assessed for repeated measures (**D**,**G**) and one-way ANOVA (**A**–**C**,**E**,**F**,**H**). CD: chow diet, HFA: HFD + d-allulose, HFC: HFD + cellulose, HFD: high-fat diet, HOMA-β: the homeostasis model assessment of β-cell function, IPGTT: intraperitoneal glucose tolerance test, and ITT: insulin tolerance test.

The fasting glucose level in the HFC group was higher than that in the other two groups (*p* = 0.052). The fasting insulin level in the HFC group was also higher than that in the other two groups (*p* < 0.05) (Figure 2A,B). No differences were observed for the value of HOMA-β between the groups (Figure 2C).

For the intraperitoneal glucose tolerance test (IPGTT), the peak was observed at approximately 320 mg/dL for the HFC group, and within the experimental period, it showed little disposal of glucose, whereas the HFA group showed significantly lower glucose levels (Figure 2D). The area under the curve (AUC) above the baseline glucose concentration illustrates that glucose uptake is ameliorated in the HFA group compared with the HFC group (Figure 2E). The basal insulin level in the HFC group was significantly higher than that in the CD group, whereas that in the HFA group was lower than that in the HFC group. At 30 min (glucose at its highest concentration), the average insulin levels in each group were higher than that at baseline. The trend of insulin levels was similar (Figure 2F).

Insulin tolerance test (ITT) analysis revealed that the HFC group had higher glucose levels than the CD group (Figure 2G). The HFA group had lower blood glucose levels than the HFC group. AUC in the HFA group was lower than that in the HFC group, suggesting improved insulin sensitivity (Figure 2H).

### 2.3. d-Allulose Ameliorates HFD-Induced Insulin Resistance

**Figure 3:**
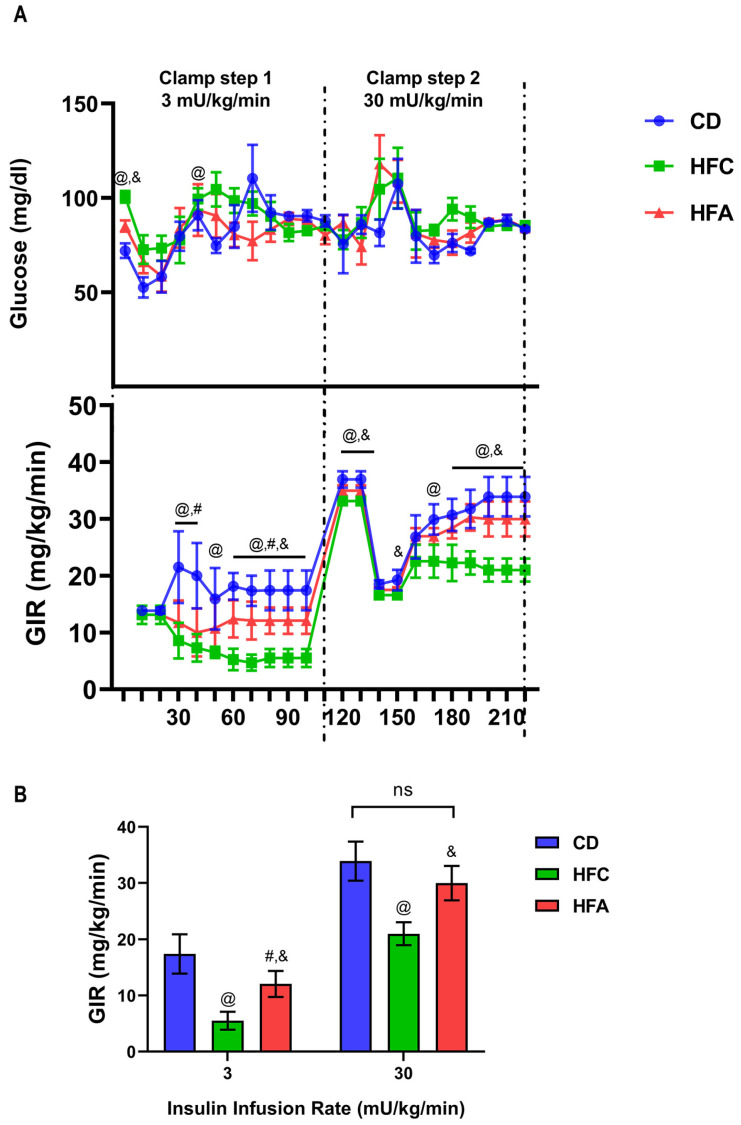
Changes in blood glucose level and glucose infusion rate (GIR) in the hyperinsulinemic–euglycemic clamp test: (**A**) The time course of glucose concentration and GIR. Low-dose insulin (3 mU/kg) was continuously infused during step 1 and high-dose insulin (30 mU/kg) during step 2; (**B**) differences in GIR at low and high insulin doses. Results are expressed as mean ± SD; n = 6 per group. @: CD vs. HFC, #: CD vs. HFA, &: HFC vs. HFA, *p* < 0.05; and ns, no significant difference. The difference was determined using one-way ANOVA assessed by each time point (**A**,**B**). CD: chow diet, HFA: HFD + d-allulose, HFC: HFD + cellulose, and HFD: high-fat diet.

To elaborate on the effect of d-allulose on insulin sensitivity, we conducted the two-step HE-clamp study. The time course of glucose concentration and glucose infusion rate (GIR) is shown in Figure 3A. Fasting glucose levels were higher in the HFC group than in the other two groups. After the first 10 min, the glucose levels of all three groups decreased rapidly, confirming that insulin was administered. GIR indicates the levels of exogenous glucose required to maintain euglycemia. In the low-dose clamp test, the GIR of the CD group was significantly higher than that of the HFC and HFA groups, whereas the GIR of the HFC group was significantly lower than that of the HFA group (*p* < 0.05) (Figure 3B). By contrast, results from the high-dose insulin clamp test revealed that the administration of d-allulose reversed the HFD-induced reduction in GIR, and there was no significant difference in GIR between the HFA and CD groups. These findings indicate that d-allulose improves systemic and muscular insulin resistance.

### 2.4. d-Allulose Supplementation Lowers TNF-α but Improves Adiponectin Level

**Figure 4:**
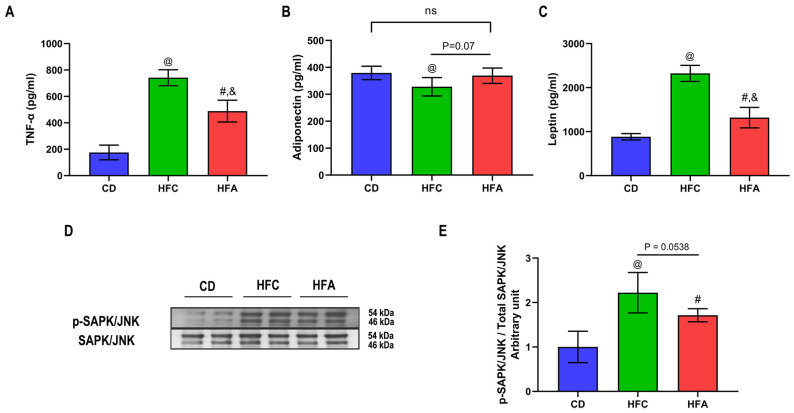
Effects of d-allulose supplementation for 4 weeks on adipokines and JNK signaling. (**A**) Levels of plasma TNF-α, (**B**) adiponectin, and (**C**) leptin. (**D**) Western blot showing bands of SAPK/JNK phosphorylation at Thr183/Tyr185 and total SAPK/JNK. (**E**) The level of SAPK/JNK phosphorylation at Thr183/Tyr185 vs. SAPK/JNK protein expression in the soleus muscle. Results are expressed as mean ± SD; n = 6 per group. @: CD vs. HFC, #: CD vs. HFA, &: HFC vs. HFA, *p* < 0.05; ns, no significant difference. The difference was determined by one-way ANOVA. CD: chow diet, HFA: HFD + d-allulose, HFC: HFD + cellulose, HFD: high-fat diet, JNK: Jun N-terminal kinase, SAPK: stress-activated protein kinase, and TNF: tumor necrosis factor.

After 4 weeks of HFD, both the HFC and HFA groups had higher plasma TNF-α levels than the CD group. In the HFA group, the level of TNF-α was significantly lower than that in the HFC group (*p* < 0.05) (Figure 4A). There was no significant difference in plasma adiponectin levels between the HFA and CD groups (Figure 4B). Plasma leptin levels increased in the HFA and HFC groups compared with the CD group (Figure 4C). The phosphorylation ratio of stress-activated protein kinase/Jun N-terminal kinase (SAPK/JNK) was significantly higher in the HFC and HFA groups than in the CD group (Figure 4D,E) but significantly lower in the HFA group than in the HFC group.

### 2.5. d-Allulose Enhances Insulin Signaling and Glucose Transporter 4 Expression

**Figure 5:**
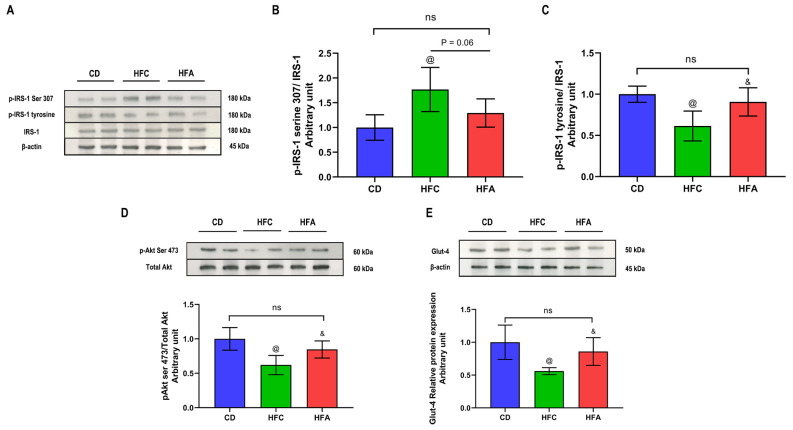
Effects of d-allulose on insulin signaling and Glut-4 expression in the soleus muscle. (**A**) Phosphorylation of IRS-1 at serine 307 and tyrosine vs. protein expression of IRS-1. (**B**) The level of insulin-stimulated phosphorylation of IRS-1 at serine 307 vs. IRS-1 protein expression. (**C**) The ratio of insulin-stimulated phosphorylation of IRS-1 at the tyrosine residue vs. IRS-1 protein expression. (**D**) The level of insulin-stimulated phosphorylation of Akt at serine 473 vs. total Akt, (**E**) Glut-4 protein expression. Results are expressed as mean ± SD; n = 6 per group, @: CD vs. HFC, &: HFC vs. HFA, *p* < 0.05; and ns, no significant difference. The difference was determined using one-way ANOVA. All the western blots used for the quantitative analysis are presented in Supplementary Figure S1. Akt: Protein kinase B, CD: chow diet, Glut-4: glucose transporter 4, HFA: HFD + d-allulose, HFC: HFD + cellulose, HFD: high-fat diet, and IRS-1: insulin receptor substrate-1.

After 4 weeks of HFD + 5% cellulose feeding, the ratio of phosphorylation of serine 307 of insulin receptor substrate-1 (IRS-1) was higher in the HFC group than in the CD group (Figure 5A,B). The ratio of phospho-IRS-1 tyrosine to IRS-1 and that of phospho-Akt (serine 473) to Akt was significantly lower in the HFC group than in the CD group (Figure 5C,D). Under d-allulose supplementation, the phosphorylation of IRS-1 (serine 307) was significantly lower in the HFA group than in the HFC group, whereas, the ratio of phospho-IRS-1 tyrosine to IRS-1 and that of phospho-Akt (serine 473) to Akt were higher in the HFA group than in the HFC group (Figure 5B–D). There was no significant difference in phosphorylation of IRS-1 at serine 307 and tyrosine vs. protein expression of IRS-1, and phospho-Akt (serine 473) to Akt between the HFA and CD groups. Finally, the expression of glucose transporter 4 (Glut-4) was higher in the HFA group than in the HFC group. There was no significant difference in Glut-4 protein expression between the HFA and CD groups (Figure 5E).

## 3. Discussion

In this study, the HE-clamp test was used to show that d-allulose supplementation ameliorated insulin resistance in the HFD group to the level in the CD group. The second step of the HE-clamp test specifically evaluates the skeletal muscle insulin resistance because the high-dose insulin infusion sufficiently suppresses hepatic gluconeogenesis. There is still a lack of direct evidence regarding the effects of d-allulose on insulin resistance in skeletal muscles. Furthermore, the study on the interplay between major insulin target tissues, muscles, liver, and fats is required to understand the underlying mechanisms of insulin resistance. We showed that d-allulose administration restored the balance between proinflammatory and anti-inflammatory adipokines in HFD-fed rats. The subsequent changes in insulin signaling, especially IRS-1, indicate that the link between the adipose tissue and skeletal muscle plays a critical role in the improvement of HFD-induced muscular insulin resistance by d-allulose in Wistar rats.

The impaired insulin signaling cascade activation and impaired Glut-4 function is the primary defect in skeletal insulin resistance [11]. In the present study, we focused on IRS-1 phosphorylation and Glut-4 expression. d-allulose increased IRS-1 tyrosine phosphorylation and decreased serine 307 phosphorylation. These results are consistent with suppressed TNF-α expression and phosphorylation of JNK and the increase in adiponectin secretion. Adiponectin reduces serine 307 phosphorylation of IRS-1 to sensitize insulin signaling in the muscle [12]. The Glut-4 expression was also increased by d-allulose administration. A study by Lee et al. demonstrated the mRNA upregulation of Glut-4, IRS-1, phosphatidylinositol-4,5-bisphosphate 3 (PI-3) kinase catalytic subunit alpha, and AKT2 in c57BL/KsJ-db/db mice [13]. In our analysis of protein expression levels, Glut-4 expression was increased by d-allulose treatment but not IRS-1 or Akt. Glut-4 translocation defects were proposed as the mechanism of insulin resistance [14]. The possibility that d-allulose may change a specific step of the insulin signaling cascade should be investigated in a future study.

We used the HE-clamp method to demonstrate for the first time that d-allulose administration ameliorates HFD-induced insulin resistance. Notably, the HE-clamp showed similar insulin sensitivity between the HFA and standard CD groups, despite the presence of higher visceral fat in the HFA group than in the CD group. Our results are consistent with those of others demonstrating that d-allulose in drinking water improves insulin sensitivity without affecting body weight or fat mass [15,16], indicating its mechanism is independent of weight loss or fat reduction. In the two-step HE-clamp test, low-dose insulin infusion GIR refers to insulin sensitivity of the liver and peripheral tissues [17], whereas high-dose insulin infusion GIR refers to the insulin sensitivity index of the skeletal muscle [18].

d-Allulose potentially ameliorates skeletal muscle insulin resistance during obesity through multiple mechanisms. First, d-allulose may reduce fat accumulation in the muscle, which has been proposed to play a role in muscle-specific insulin resistance [19]. Second, d-allulose may reduce mitochondrial dysfunction [15], thereby ameliorating insulin resistance. d-Allulose supplementation may have similar effects in the skeletal muscle [20]. Finally, d-allulose may increase insulin-mediated endothelial NO production to improve insulin-mediated microvascular blood flow, thereby enhancing muscle glucose uptake [21]. However, further research is required to clarify the mechanism of how d-allulose improves muscle insulin resistance.

Adipose tissue is primarily responsible for the development of insulin resistance caused by HFD. The balance between proinflammatory and anti-inflammatory adipokines plays a role in insulin resistance. Our findings are consistent with those of others in that d-allulose could improve the metabolic function in adipose tissue. Moreover, d-allulose significantly suppressed TNF-α expression and phosphorylation of JNK. d-Allulose was reported to reduce the accumulation of macrophages around inflamed adipocytes and suppress the generation of reactive oxygen species, thereby reducing the release of proinflammatory cytokines [22]. By contrast, d-allulose promoted the secretion of adiponectin, which could be because d-allulose ameliorated high-fat diet-induced adipocyte hypertrophy [23,24]. Thus, d-allulose could directly ameliorate systemic insulin resistance by suppressing proinflammatory cytokine production and promoting adiponectin secretion. Moreover, improving adipose tissue metabolism could contribute to the enhancement of muscle insulin resistance specifically [13]. TNF-α production and JNK phosphorylation could directly act on IRS-1 signaling in the muscle, and adiponectin reduced serine 307 phosphorylation of IRS-1 to sensitize insulin signaling in the muscle [12].

d-Allulose promotes energy expenditure to prevent weight gain and obesity. Studies have shown that d-allulose increases β-oxidation [25] and energy expenditure [26]. Consistently, our data revealed that under the same caloric intake, the body weight and the feed efficiency rate were lower in the HFA group than in the HFC group. Taken together, it indicates that d-allulose increased energy expenditure. Moreover, increased energy expenditure could contribute to suppressed fat accumulation [27], which may explain why the HFA group had lower visceral fat than the HFC group.

This study has certain limitations. Hepatic insulin resistance may crosstalk with muscle insulin resistance. Although many studies have focused on the effect of d-allulose on the liver in insulin resistance [8,28,29,30], we did not focus on this aspect in this study. We also used only the soleus muscle for muscle insulin signaling analysis. Although Albers et al. have reported that type I muscles and type II muscles have a similar sensitivity for phosphoregulation by insulin [31], different muscles might respond differently to insulin. Moreover, we fed the rats each diet for 4 weeks and observed the differences in body weight and visceral fat between the groups. These factors are known to affect insulin sensitivity in the skeletal muscle indirectly. Further studies on evaluating the bodyweight of paired subjects using the HE-clamp method need to be conducted by excluding influencing factors discussed in the preceding sections.

In summary, this is the first report to demonstrate that d-allulose possesses protective effects against HFD-induced systemic and skeletal muscle insulin resistance using the HE-clamp method. Consistently, d-Allulose inhibited the phosphorylation of IRS-1 and enhanced Akt phosphorylation at serine 307 in the skeletal muscle. The suppression of TNF-α and JNK signaling pathways and enhancement of glucose uptake and adiponectin secretion could contribute to the protective effects of d-allulose against insulin resistance. These findings suggest that d-allulose can be developed into functional food against insulin resistance associated with obesity.

## 4. Materials and Methods

### 4.1. Animals and Diets

Six-week-old male Wistar rats were purchased from Chubu Kagakusizai Co., Ltd. (Aichi Prefecture, Japan). During the experimental period, rats were individually housed at a constant temperature of 22 ± 2 °C under artificial lighting (12:12-h, 8 am-8 pm, reverse light-dark cycle), with access to food and water ad libitum. The rats were acclimatized for two weeks and then randomly divided into three groups: CD (Oriental Yeast Co., Ltd., Tokyo, Japan), HFC, or HFA. Rats in the HFA group were fed HFD containing 5% (*w*/*w*) d-allulose (Matsutani Chemical Industry Co., Ltd., Hyogo Prefecture, Japan). Rats in the HFC group were fed HFD plus 5% (*w*/*w*) cellulose (Oriental Yeast Co., Ltd., Tokyo, Japan) as a substitute for d-allulose. The nutritional profile (*w*/*w*) of each diet is as follows: CD; 23.6% protein, 5.3% fat, and 57.3% carbohydrates (energy percentage: protein 22.7%, fat 11.1%, and carbohydrates 66.2%). HSC and HSA: 23.1% protein, 34.9% fat, 6.5% fiber, and 25.9% carbohydrates (energy percentage: protein 18.1%, fat 61.6%, carbohydrates 20.3%). All experimental procedures followed the Guidelines for the Care and Use of Laboratory Animals of Nagoya University. Ethical approval was granted by the Animal Experiment Committee of Nagoya University (HPFS No. 19).

### 4.2. IPGTT and ITT

After 4 weeks of diet administration, rats were fasted from the end of the light period (8 am) for 6 h before IPGTT and injected with a bolus of 20% glucose (2 g/kg, i.p.). Blood samples were collected from the tail vein at the following time points: T = 0 (before glucose injection) and 30, 60, 90, and 120 min after glucose injection to evaluate glucose concentrations. Time 0 and 30 min blood samples were taken for insulin measurement.

For ITT, rats were fasted from the beginning of the light period (8 pm) for 12 h and injected with 0.75 U insulin (i.p.). Blood samples were collected from the tail vein at 0 (before insulin injection), 30, 60, 90, and 120 min. Blood glucose levels were measured for each time interval during ITT. Data (obtained glucose levels) were plotted as graphs, and AUC was calculated.

### 4.3. Blood Glucose and Plasma Insulin

Blood was obtained from the tail veins after 3 h fasting from the end of the light period (8 am). Blood glucose level was recorded using the blood glucose meter (BF-5S, Oji Scientific Instruments Co., Ltd., Hyogo, Japan). Plasma insulin level was measured using the ELISA kit (FUJIFILM Wako Sibayagi Corporation, Gunma, Japan) and the Multiskan FC device (Thermo Fisher Inc., Massachusetts, USA). HOMA-β value was calculated by the following formula: 360 × fasting insulin (μU/mL)/(fasting glucose [mg/dL] − 63).

### 4.4. HE-Clamp Study

The operations for cannulation and the HE-clamp test were performed as described previously [9,32]. After 4 weeks of being fed the respective diets, the rats were anesthetized with three anesthetics (medetomidine, 0.15 mg/kg; midazolam, 2 mg/kg; and butorphanol, 2.5 mg/kg i.p.) before the operation. The right jugular vein (for 20% glucose solution and insulin infusion) and the left carotid artery (for blood sampling) were catheterized. After about one week, the HE-clamp test was performed, when their bodyweight loss was <10% that before the operation. The rats were fasted from the beginning of the light period (8 pm) for 16 h, kept awake, and allowed to move freely during the test. Blood samples were obtained for measuring the basal blood glucose level after loading the infusion system for about 30 min. We conducted a continuous two-step clamp study. In the first step, insulin was infused at 3 mU/kg BW/min (low-dose, 0–100 min). In the second step, insulin was infused at 30 mU/kg BW/min (high-dose, 110–220 min). During the clamp test, the time interval of blood glucose measurement was every 10 min, and glucose infusion was adjusted to maintain euglycemia (90 mg/dL). The steady-state level was determined when the last three consecutive blood glucose changes were maintained within 18 mg/dL. GIR at the steady was regarded as the index of insulin resistance.

### 4.5. Plasma Cytokines and Adipokines

Blood samples were collected in heparinized Hematocrit Capillary Tubes (Belden Co., Ltd., St. Louis, MO, USA) and centrifuged for 10 min (4 °C, 2000× *g*). Plasma samples were aliquoted and stored at −80 °C. Plasma cytokine levels were determined using three detection kits from Abcam Co., Ltd., Cambridge, United Kingdom (TNF-α: GR3365123-1, leptin: GR3382338-2, and adiponectin: GR3382297-1), according to the manufacturer’s protocols.

### 4.6. Inflammatory and Insulin Signaling Analysis in Skeletal Muscle using Western Blotting

Insulin (0.5 IU/kg) was injected into the inferior vena cava, and the soleus muscle was harvested after 2 min [9]. Samples were snap-frozen in liquid nitrogen and stored at −80 °C for Western blotting.

Western blotting was performed as described previously [33]. In brief, 20 μg protein extracts from muscle tissue were separated by SDS-PAGE and transferred to polyvinylidene difluoride membranes. The membranes were blocked for 1 h at 20–25 °C with 5% nonfat milk in TBS-T buffer (20 mM Tris; pH 7.6, 0.8% NaCl, and 0.1% Tween 20) and incubated overnight at 4 °C with the following primary antibodies: phospho-Akt (serine 473) (Lot: 31942), β-actin (13E5) (Lot: 4970), phospho-SAPK/JNK (Thr183/Tyr185) (Lot: 4668), SAPK/JNK (Lot: 9252), IRS-1 (Lot: 2382), and phospho-IRS-1(serine 307) (Lot: 2381) (all from Cell Signaling Technology, Inc., Danvers, USA); Akt1/2/3 (H-136): sc-8312 and anti-phospho-Tyr antibody (PY99): sc-7020 (both from Santa Cruz Biotechnology, Inc., Santa Cruz, California, USA); and the antiglucose transporter 4 antibody (Lot: 4670-1704) (from Bio-Rad Laboratories, Inc., California, USA). Membranes were washed five times in TBS-T for 5 min each, incubated in a 1:3000 dilution of horseradish peroxidase-conjugated goat antirabbit (Bio-Rad, Laboratories Inc., Hercules, CA, USA) or antimouse (KPL, Gaithersburg, MD, USA) IgG antibody for one hour at room temperature, and washed five times in TBS-T. Protein bands were detected using the ECL reagent (GE Healthcare UK Ltd., Buckinghamshire, UK). Images of each membrane were taken on film and analyzed using the ImageJ software (National Institutes of Health, Bethesda, MD, USA).

Individual CD group data points were divided by the group mean. Thus, the mean of the normalized CD group is 1 with variability. The density of the protein band of the HFC and HFA groups was expressed as the fold change in density of CD group values.

### 4.7. Statistical Analysis

All data are expressed as the mean ± SD. Data from the longitudinal time course studies were analyzed using two-way ANOVA assessed for repeated measures (with Tukey’s HSD post hoc test). Differences between the three groups were determined using one-way ANOVA (with Tukey’s HSD post hoc test). Significance was accepted at *p* < 0.05. All analyses were performed using GraphPad Prism 8.0 (GraphPad Software Inc., San Diego, CA, USA).

## Data Availability

The data presented in this study are available on request from the corresponding author.

## Supplementary Materials

The following are available online, Figure S1: Western blots used for the quantitative analysis. Table S1: skeletal muscle masses.

## Sample Availability

Samples of the compounds are not available from the authors.
