# Supplementary material for: d-Allulose Ameliorates Skeletal Muscle Insulin Resistance in High-Fat Diet-Fed Rats
Source: Molecules. 2021 Oct 19;26(20):6310. doi: 10.3390/molecules26206310 (PMC8539500; doi:10.3390/molecules26206310)
Supplement: Supplementary file 1 [file molecules-26-06310-s001.zip › molecules-1385374-SI.pdf]

Supplementary Figure S1

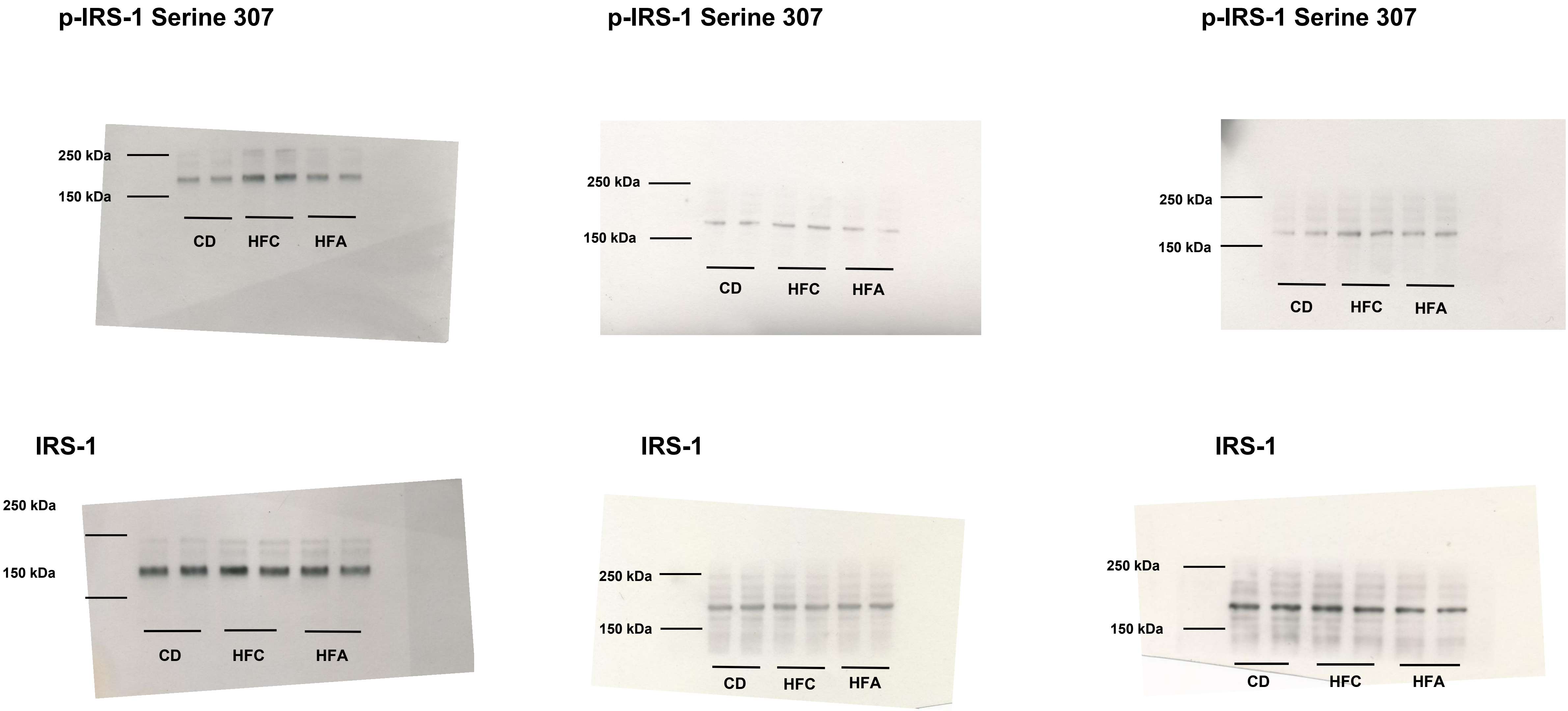

The western blot used for display and the quantitative analysis in Figure 5A are shown.

Supplementary Figure 1

p-IRS-1 tyrosine

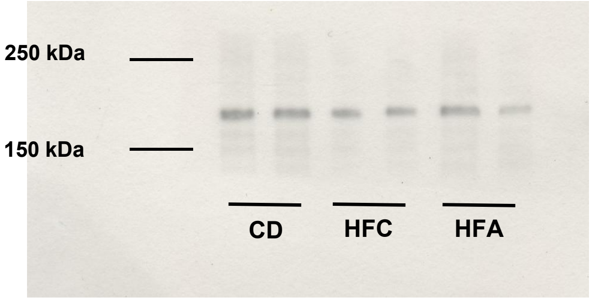

p-IRS-1 tyrosine

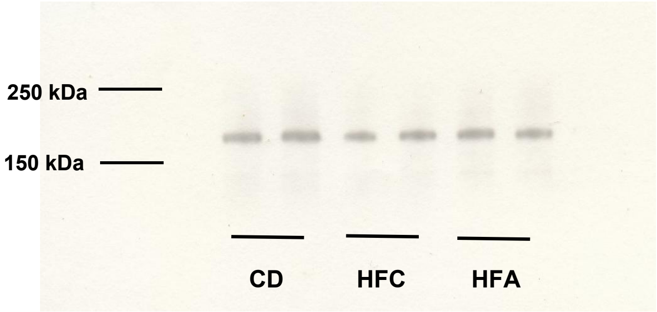

p-IRS-1 tyrosine

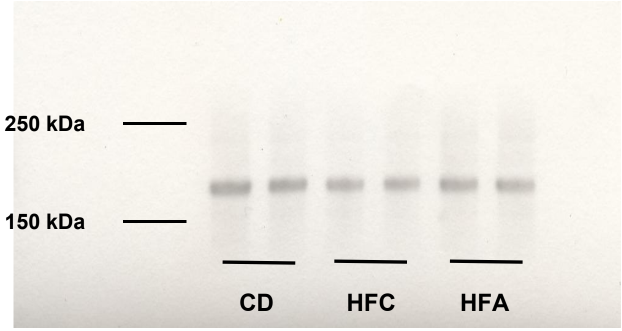

p-IRS-1 tyrosine

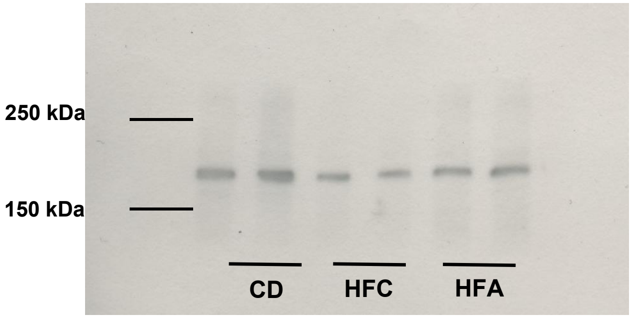

The western blot used for display and the quantitative analysis in Figure 5A are shown.

Supplementary Figure 1

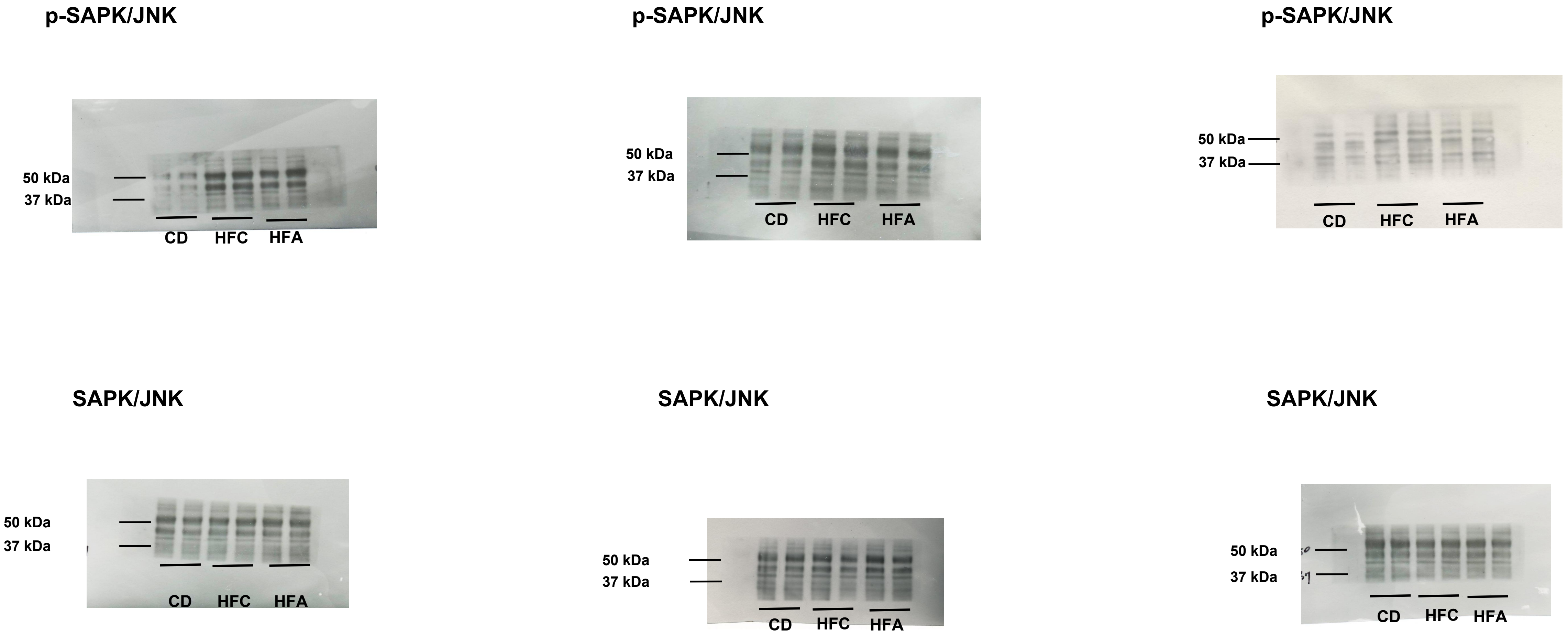

The western blot used for display and the quantitative analysis in Figure 4D are shown.

Supplementary Figure 1

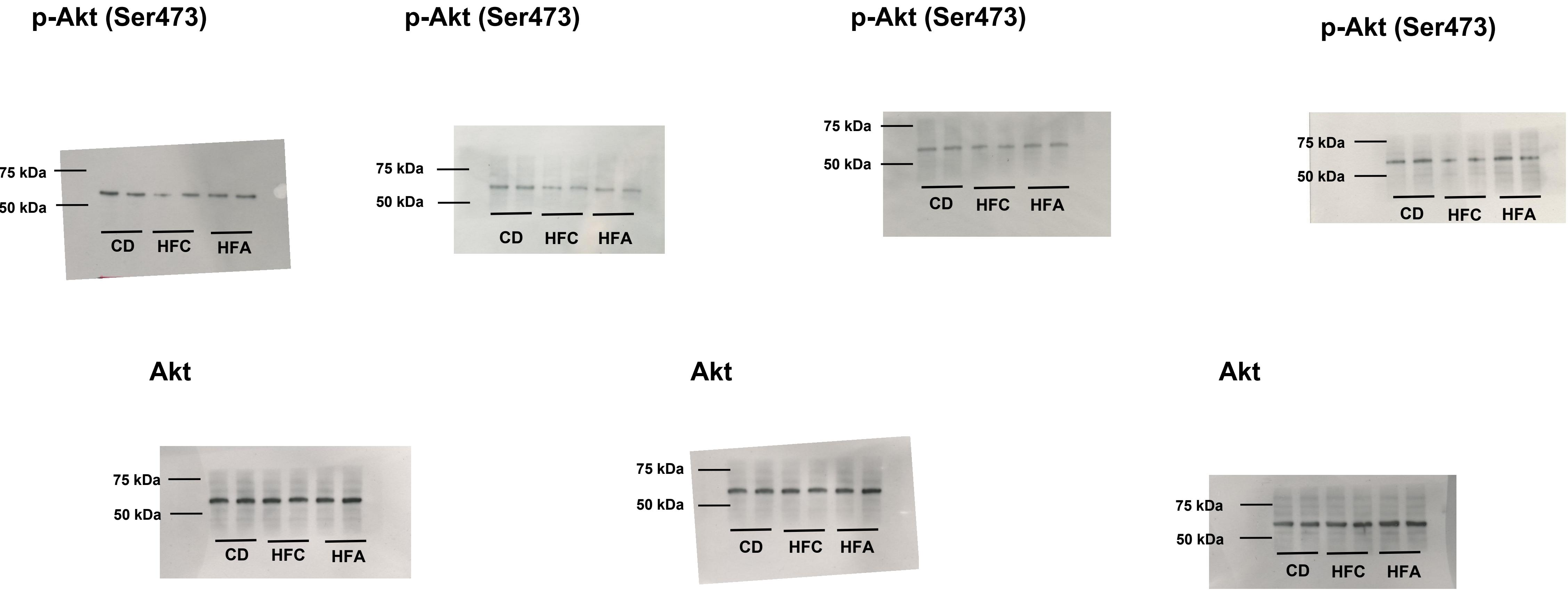

The western blot used for display and the quantitative analysis in Figure 5D are shown.

Supplementary Figure 1

Glut-4

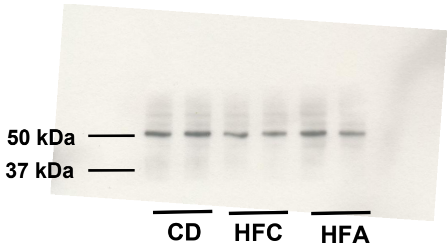

Glut-4

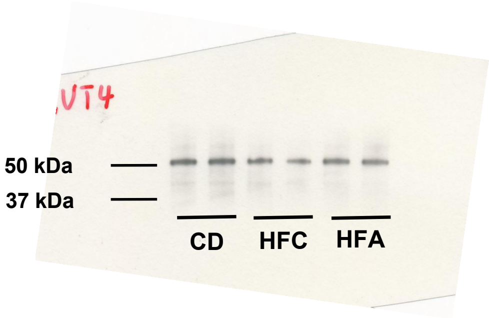

Glut-4

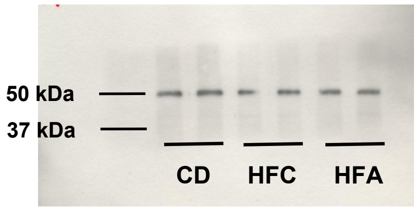

Glut-4

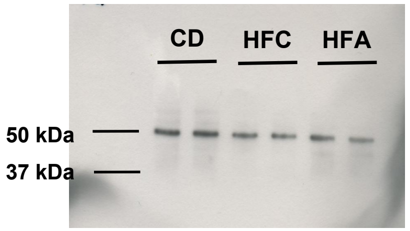

β-actin

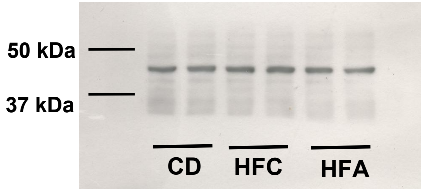

β-actin

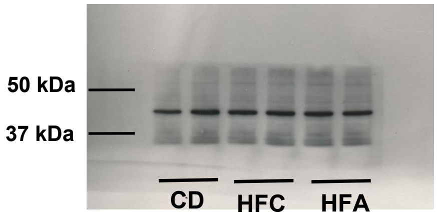

β-actin

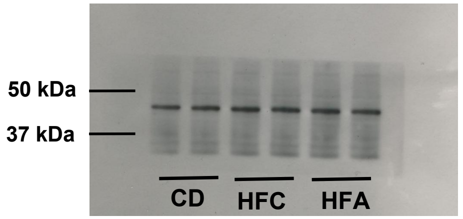

The western blot used for display and the quantitative analysis in Figure 5E are shown.

Supplementary Table S1

Muscle mass (g)	CD	HFC	HFA
Gastrocnemius	2.024 ± 0.433	1.894 ± 0.286	1.931 ± 0.182
Plantaris	0.375 ± 0.070	0.392 ± 0.051	0.394 ± 0.059
Soleus	0.128 ± 0.011	0.104 ± 0.016[@]	0.119 ± 0.018
Tibialis anterior muscle	0.591 ± 0.069	0.539 ± 0.080	0.590 ± 0.160
Extensor digitorium longus	0.229 ± 0.027	0.231 ± 0.043	0.220 ± 0.026

Table S1. Effects of D-allulose supplementation for 4 weeks on skeletal muscle mass. Results are expressed as mean ± SD; n = 6 per group, @: CD vs. HFC. CD: chow diet, HFC: HFD + cellulose, HFA: HFD + D-allulose.
